# Patient-Reported Palliative Care Outcomes in Kazakhstan: A Cross-Regional Study Using the POS Questionnaire

**DOI:** 10.3390/healthcare14142131

**Published:** 2026-07-16

**Authors:** Aigerim Mukusheva, Zaituna Khismetova, Sholpan Tokesheva, Balday Issenova, Gulnaz Kairatova, Zhanara Buribayeva, Kamila Akhmetova, Nazym Iskakova

**Affiliations:** 1 Department of Public Health, Semey Medical University, Semey 071407, Kazakhstan; aigerim.mukusheva@smu.edu.kz (A.M.); zaituna.khismetova@smu.edu.kz (Z.K.); sholpan.tokesheva@smu.edu.kz (S.T.); 2 Department of Nursing, Kazakh National Medical University, Almaty 050000, Kazakhstan; isenova.b@kaznmu.kz (B.I.); buribaeva.zh@kaznmu.kz (Z.B.); 3 Department of Public Health and Management, Astana Medical University, Astana 010000, Kazakhstan; akhmetova.km@amu.kz

**Keywords:** palliative care, patient-reported outcomes, Palliative Outcome Scale, POS, quality of life, symptom burden, Kazakhstan, cross-sectional study

## Abstract

**Background:** Patient-reported outcome measures are increasingly used to evaluate the quality of palliative care. However, evidence from Kazakhstan remains limited. This study aimed to assess palliative care outcomes among adult patients in Kazakhstan using the Palliative Outcome Scale (POS). **Methods:** A cross-sectional study was conducted among 134 adult patients receiving palliative care in East Kazakhstan. Data were collected using a socio-demographic and clinical questionnaire and the 10-item POS. Descriptive statistics, Cronbach’s alpha, Spearman correlation, nonparametric tests, and multiple linear regression were performed using IBM SPSS Statistics 26.0. **Results:** The mean total POS score was 10.13 ± 5.05, indicating a moderate burden of palliative care-related problems. The highest mean scores were observed for self-worth, meaning in life, anxiety, and pain. The POS demonstrated moderate internal consistency (Cronbach’s α = 0.641). Significant differences in total POS scores were found across disease profiles (*p* < 0.001), whereas no significant differences were observed by gender or age. In multiple linear regression, disease profile was the only variable significantly associated with the total POS score (B = 0.688, β = 0.292, *p* = 0.001). **Conclusions:** Patients receiving palliative care in Kazakhstan experience a moderate burden of physical, emotional, and existential concerns. Disease profile was significantly associated with patient-reported outcomes and may represent one of several factors influencing palliative care needs. The POS may be a useful tool for assessing patient-reported palliative care outcomes in Kazakhstan; however, further formal psychometric validation in the local population is required before routine implementation.

## 1. Introduction

Palliative care is an essential component of health systems aimed at reducing suffering and improving quality of life for people with life-threatening illnesses. Globally, more than 56.8 million people require palliative care each year, and this number is projected to rise substantially by 2060, particularly in low- and middle-income countries [1,2,3,4]. Early integration of palliative care has been shown to improve quality of life and emotional well-being among patients with advanced cancer [5,6].

Patient-reported outcome measures (PROMs) are increasingly used to assess symptoms and concerns directly from the patient’s perspective and to evaluate the effectiveness of care [7,8,9]. The Palliative Outcome Scale (POS) is one of the most widely used multidimensional instruments, covering physical, psychological, social, informational, and spiritual domains [10]. In addition to symptom burden assessment, the POS can also be considered a multidimensional measure of quality of life in palliative care, as it evaluates physical, psychosocial, emotional, informational, and existential aspects of patient well-being. Its extended version, the Integrated Palliative care Outcome Scale (IPOS), has demonstrated good psychometric properties across various populations and languages [11,12,13,14,15]. Although the Integrated Palliative care Outcome Scale (IPOS) has gained wider international application in recent years, the original POS remains one of the most established multidimensional instruments in palliative care and allows comparability with earlier foundational studies. In the present study, POS was selected due to its concise structure, feasibility of administration, and suitability for exploratory assessment in patients with advanced illness.

Despite their value, PROMs remain underused in many countries, and concerns have been raised regarding their ability to capture culturally specific and relational aspects of suffering [16,17]. In Kazakhstan, where the burden of cancer is substantial, palliative care services continue to face challenges such as limited access, insufficient training, and shortages of essential medicines [18,19,20,21]. However, despite several studies addressing palliative care organization and access in Kazakhstan, there remains very limited evidence regarding patient-reported symptom burden, psychosocial distress, existential concerns, and quality-of-life outcomes assessed using standardized multidimensional instruments. In Kazakhstan, the routine implementation of standardized patient-reported outcome measures in palliative care remains limited. Clinical assessment is still largely based on physician-reported observations and symptom-oriented evaluation, while structured multidimensional patient-centered tools are not yet widely integrated into routine practice. Similar challenges in implementing patient-reported palliative care outcome measures have also been reported in other low- and middle-income countries and neighboring regions, where limited resources, insufficient staff training, and lack of validated local instruments remain important barriers.

This study aimed to assess palliative care outcomes among adult patients in Kazakhstan using the POS and to examine their associations with socio-demographic and clinical characteristics.

## 2. Materials and Methods

### 2.1. Study Design and Setting

This cross-sectional descriptive study was conducted to assess patient-reported palliative care outcomes among adult patients receiving palliative care in the East Kazakhstan region. Data collection was carried out from September 2025 to March 2026 in specialized palliative care departments and healthcare institutions providing inpatient and outpatient palliative services. The study was designed to evaluate physical, psychological, social, informational, and existential concerns using the Palliative Outcome Scale (POS) and to identify socio-demographic and clinical factors associated with the total POS score.

### 2.2. Study Population and Eligibility Criteria

The target population consisted of adult patients receiving palliative care for life-limiting illnesses. Eligible participants were men and women aged 18 years and older who had a confirmed diagnosis of an advanced chronic or progressive disease, were receiving palliative care at the time of the study, were able to understand the purpose and content of the questionnaire, and provided written informed consent.

Patients were included regardless of their underlying diagnosis, including cancer and non-cancer conditions such as stroke, dementia, Parkinson’s disease, dyscirculatory encephalopathy, diabetes mellitus, and bronchial asthma. The inclusion of heterogeneous diagnostic groups reflects the real-world diversity of patients receiving palliative care services and allows broader assessment of multidimensional needs across different disease trajectories. A total of 145 eligible patients were approached during the study period. Of these, 134 agreed to participate (response rate: 92.4%), while 11 declined participation. The most common reasons for refusal included fatigue, emotional distress, and unwillingness to participate. Patients were excluded if they were younger than 18 years, had severe communication difficulties preventing meaningful self-reporting and questionnaire completion, had severe cognitive impairment preventing meaningful participation, were in a critical clinical condition, or declined to participate.

### 2.3. Sample Size and Sampling Method

A non-probability convenience sampling method was used. All patients who met the inclusion criteria and were available during the study period were invited to participate. Recruitment continued until the planned sample size was achieved.

A total of 134 patients were enrolled and completed the questionnaire. The sample size was determined based on feasibility, institutional patient flow, and methodological recommendations for regression analyses, which generally require at least 10–15 observations per predictor variable. Given the four main predictors included in the model, the final sample of 134 participants was considered sufficient. The sample included patients with a broad range of diagnoses and demographic characteristics, which allowed for a comprehensive assessment of palliative care outcomes across different clinical groups.

### 2.4. Data Collection Procedure

Data were collected through structured face-to-face interviews conducted by trained researchers and healthcare professionals familiar with palliative care. Before the interview, each participant received verbal and written information describing the objectives of the study, the voluntary nature of participation, and the confidentiality of responses. Written informed consent was obtained from all participants prior to enrollment.

Participants completed the questionnaire in paper format. Depending on their physical condition, literacy level, and personal preference, the questionnaire was completed independently, with assistance from a relative or caregiver, or with support from a healthcare professional. For participants who required assistance, standardized instructions were used, and no interpretation or modification of questions was permitted in order to minimize interviewer influence and social desirability bias. The average time required to complete the questionnaire ranged from 10 to 15 min. Completed questionnaires were reviewed for completeness and coded for subsequent statistical analysis.

### 2.5. Study Instrument

The study instrument consisted of two sections: (1) a socio-demographic and clinical questionnaire developed by the researchers, and (2) the Palliative Outcome Scale (POS), a validated multidimensional instrument widely used in palliative care research and clinical practice.

#### 2.5.1. Socio-Demographic and Clinical Questionnaire

The first section of the questionnaire collected information on key participant characteristics, including sex, age group, primary diagnosis, and the method used to complete the questionnaire. Sex was categorized as male or female. Age was grouped into four categories: 18–34 years, 35–49 years, 50–64 years, and 65 years and older. Diagnosis was recorded according to the patient’s primary medical condition. The questionnaire completion method was categorized as self-completed, completed with assistance from a relative or caregiver, or completed with assistance from a healthcare professional.

These variables were used to describe the study population and to examine their relationship with the total POS score.

#### 2.5.2. Palliative Outcome Scale (POS)

The Palliative Outcome Scale (POS) is a standardized and validated patient-reported outcome measure developed by Hearn and Higginson to assess core outcomes in palliative care [10]. In this study, the Russian-language version of the POS questionnaire was used, as Russian remains one of the primary languages of clinical communication in Kazakhstan. Prior to data collection, the questionnaire was reviewed by palliative care specialists for linguistic clarity and contextual appropriateness. Although formal psychometric validation has not yet been conducted in the Kazakhstani population, a pilot assessment was carried out to evaluate the comprehensibility and feasibility of administration. The instrument contains 10 items addressing the following domains:

1. Pain experienced during the previous three days;
2. Other symptoms, such as nausea or constipation;
3. Patient anxiety and worry;
4. Anxiety and worry among family members or close friends;
5. Adequacy of information provided to the patient;
6. Ability to share feelings with family or friends;
7. Sense that life is worthwhile;
8. Feeling good about oneself as a person;
9. Practical problems resulting from illness;
10. Time wasted in obtaining medical care.

Each item is scored on a 5-point Likert scale ranging from 0 to 4. Higher scores indicate greater symptom burden, psychosocial distress, or unmet needs. The total POS score is calculated by summing all ten items and ranges from 0 to 40, with higher scores reflecting poorer palliative care outcomes.

The POS has been translated and validated in multiple languages and has demonstrated acceptable psychometric properties across diverse populations and healthcare settings. In the present study, the internal consistency of the instrument was assessed using Cronbach’s alpha.

### 2.6. Variable Definitions

The primary dependent variable was the total POS score (POS_total), calculated as the sum of the scores for all ten POS items. This variable was treated as a continuous outcome measure representing the overall burden of palliative care-related problems.

Independent variables included:

- Gender (male, female);
- Age group (18–34, 35–49, 50–64, and ≥65 years);
- Disease profile (categorized according to the primary diagnosis);

Questionnaire completion method (self-completed, with assistance from a relative, or with assistance from a healthcare professional).

In addition, each individual POS domain was analyzed separately to describe the distribution of physical, psychological, social, informational, and existential concerns and to examine correlations with the total POS score.

### 2.7. Statistical Analysis

Statistical analysis was performed using IBM SPSS Statistics version 26.0 (IBM SPSS Statistics). Descriptive statistics were used to summarize participants’ socio-demographic and clinical characteristics. Categorical variables were presented as frequencies and percentages, while continuous variables were reported as means and standard deviations.

The internal consistency of the Palliative Outcome Scale (POS) was assessed using Cronbach’s alpha coefficient. Correlations between individual POS domains and the total POS score were examined using Spearman’s rank correlation coefficient.

The normality of variable distributions was assessed using the Kolmogorov–Smirnov and Shapiro–Wilk tests. Because the majority of variables did not follow a normal distribution, non-parametric methods were applied for group comparisons. Differences between two independent groups were analyzed using the Mann–Whitney U test, while comparisons among more than two groups were conducted using the Kruskal–Wallis test.

Prior to regression analysis, key assumptions, including normality of residuals, homoscedasticity, linearity, and independence of errors, were assessed and found to be acceptable. Disease profile was entered into the regression model as an ordinal-coded variable representing the primary diagnosis category. To identify factors associated with the total POS score, multiple linear regression analysis was performed. The total POS score was used as the dependent variable, and gender, age group, disease profile, and questionnaire completion method were entered as independent variables. Regression coefficients (B), standardized beta coefficients (β), 95% confidence intervals, and *p*-values were reported. Multicollinearity was assessed using variance inflation factors (VIF). Statistical significance was defined as *p* < 0.05.

### 2.8. Ethical Considerations

The study was conducted in accordance with the ethical principles outlined in the Declaration of Helsinki and relevant national regulations governing biomedical research involving human participants. Ethical approval for the study was obtained from the Local Ethics Committee of Semey Medical University (Protocol No. 2, dated 5 December 2024).

Prior to participation, all eligible patients were informed about the objectives of the study, the voluntary nature of participation, and their right to withdraw at any time without any consequences for their medical care. Written informed consent was obtained from all participants before data collection.

Confidentiality and anonymity were strictly maintained throughout the study. Personal identifiers were not recorded in the study database, and all completed questionnaires were assigned unique identification numbers. Access to the data was restricted to the research team, and the results are presented in aggregate form only. No financial incentives were provided to participants.

## 3. Results

### 3.1. Participant Characteristics 

A total of 134 palliative care patients were included in the study. The sample consisted of 60 male patients (44.8%) and 74 female patients (55.2%). Most participants were aged 65 years and older (61.2%), followed by those aged 50–64 years (27.6%).

The most common diagnoses were dyscirculatory encephalopathy (25.4%), cancer (20.1%), and stroke (18.7%). Other diagnoses included Parkinson’s disease (11.2%), dementia (10.4%), diabetes mellitus (9.7%), bronchial asthma (3.0%), and syphilis (1.5%).

Regarding questionnaire completion, nearly half of the respondents (45.5%) completed the questionnaire with the assistance of a staff member, while 27.6% completed it independently and 26.9% received help from a friend or relative. Detailed sociodemographic and clinical characteristics are presented in Table 1.

### 3.2. Descriptive Statistics of Palliative Care Outcome Scale Scores

The mean total Palliative care Outcome Scale (POS) score was 10.13 ± 5.05 (observed range: 1–24), indicating a moderate overall symptom burden and psychosocial distress among palliative care patients.

Among the individual POS domains, the highest mean scores were observed for feeling good about oneself (1.89 ± 1.21) and feeling that life is worthwhile (1.83 ± 1.22), indicating greater existential distress and unmet psychosocial needs related to self-worth and meaning in life. Higher scores were also reported for anxiety and worry during the last three days (1.43 ± 1.24) and difficulties in sharing feelings with family (1.37 ± 1.42). Lower mean scores were found for receiving adequate information (0.33 ± 0.79), practical problems (0.34 ± 0.83), and time wasted due to healthcare visits (0.15 ± 0.53), suggesting that these issues were less burdensome for most participants. Detailed descriptive statistics are presented in Table 2.

### 3.3. Reliability of the POS Questionnaire

The internal consistency of the Palliative care Outcome Scale (POS) was assessed using Cronbach’s alpha. The Cronbach’s alpha coefficient of 0.641 indicated moderate internal consistency. Although this level of reliability may be considered acceptable for exploratory research, it suggests that further psychometric evaluation of the POS is required before broader implementation in the local context. The reliability analysis of the POS questionnaire is presented in Table 3. The table summarizes the internal consistency coefficients obtained for the instrument.

### 3.4. Correlation Analysis of POS Domains

Spearman’s rank correlation analysis demonstrated statistically significant associations between several POS domains and the total POS score. The strongest positive correlations with the total score were observed for feeling that life is worthwhile (r = 0.775, *p* < 0.001) and feeling good about oneself (r = 0.745, *p* < 0.001). Moderate positive correlations were also found for pain (r = 0.618, *p* < 0.001), anxiety and worry (r = 0.598, *p* < 0.001), and the ability to share feelings with family (r = 0.530, *p* < 0.001).

Weaker but statistically significant correlations were identified for family anxiety (r = 0.355, *p* < 0.001), nausea and constipation (r = 0.313, *p* < 0.001), and practical problems (r = 0.225, *p* = 0.009). Time wasted due to healthcare visits showed a weak negative correlation with the total POS score (r = −0.185, *p* = 0.032). No statistically significant correlation was found between the adequacy of information received and the total POS score (r = 0.159, *p* = 0.066).

Since the total POS score includes all individual domains, these correlations should be interpreted cautiously because part-whole overlap may have contributed to the strength of these associations.

These findings suggest that psychological and existential domains, particularly self-worth and meaning in life, showed the strongest correlations with the total POS score. However, these associations should be interpreted cautiously because these domains contribute directly to the calculation of the total POS score. Table 4 presents the Spearman correlation coefficients between individual POS domains and the total POS score, illustrating the strength and direction of the observed associations.

### 3.5. Comparison of Total POS Scores Across Participant Groups

The total POS score did not differ significantly between male and female participants. According to the Mann–Whitney U test, the mean rank was 64.14 for men and 70.22 for women, and the difference was not statistically significant (U = 2018.5, Z = −0.904, *p* = 0.366).

Similarly, no statistically significant differences in total POS scores were observed across age groups. The Kruskal–Wallis test showed that participants aged 18–34, 35–49, 50–64, and 65 years and older had comparable scores (H = 1.321, df = 3, *p* = 0.724).

In contrast, statistically significant differences were found across diagnostic groups (H = 28.356, df = 7, *p* < 0.001). Participants with cancer had the highest mean rank (89.91), followed by those with syphilis (84.00), diabetes mellitus (77.00), bronchial asthma (74.50), and Parkinson’s disease (74.77). The lowest mean rank was observed among patients with dementia (28.46). However, some diagnostic subgroups, particularly bronchial asthma and syphilis, had very small sample sizes, which may limit the robustness and interpretability of subgroup comparisons. These findings suggest that disease profile may represent one of several factors associated with variation in overall palliative care outcomes. Table 5 presents the mean ranks of total POS scores across the different diagnostic groups included in the study. Post hoc analyses were not performed following the Kruskal–Wallis test; therefore, the specific between-group differences should be interpreted cautiously. Table 6 summarizes the results of the nonparametric comparisons of total POS scores according to participants’ demographic and clinical characteristics.

### 3.6. Factors Associated with the Total Palliative Outcome Scale Score

A multiple linear regression analysis was conducted to identify socio-demographic and clinical factors associated with the total Palliative Outcome Scale (POS) score. The dependent variable was the total POS score, while gender, age group, disease profile, and method of questionnaire completion were included as independent variables.

The overall regression model was statistically significant (F = 3.277, *p* = 0.014), indicating that the selected variables were jointly associated with the total POS score. The model explained 9.2% of the variance in POS scores (R^2^ = 0.092; adjusted R^2^ = 0.064).

Among the evaluated predictors, disease profile was the only variable significantly associated with the total POS score (B = 0.688, β = 0.292, *p* = 0.001; 95% CI: 0.278–1.097). This finding indicates that the type of underlying disease had a significant influence on patients’ palliative care outcomes.

Gender (*p* = 0.215), age group (*p* = 0.497), and method of questionnaire completion (*p* = 0.226) were not significantly associated with the total POS score.

No evidence of multicollinearity was observed, with variance inflation factor (VIF) values ranging from 1.009 to 1.120. Table 7 summarizes the results of the multiple linear regression analysis examining the associations between participant characteristics and the total POS score.

## 4. Discussion

This study provides one of the first quantitative patient-reported assessments of palliative care outcomes in Kazakhstan using the Palliative Outcome Scale (POS). The findings demonstrated a moderate overall burden of physical, psychological, social, and existential concerns among patients receiving palliative care, with a mean total POS score of 10.13 ± 5.05. Among the evaluated factors, disease profile was identified as one of the significant factors associated with variation in total POS scores, whereas gender, age group, and questionnaire completion method were not significantly associated with patient-reported outcomes. Although the questionnaire completion method was not significantly associated with total POS scores, assisted completion may have introduced response bias despite standardized administration procedures. However, the relatively low proportion of explained variance suggests that other important determinants of patient-reported outcomes were not captured in the present model.

The observed total POS score suggests that palliative care patients in Kazakhstan experience a substantial level of unmet needs. This finding is consistent with international studies demonstrating that patients with advanced illness frequently report persistent symptoms and psychosocial distress despite receiving palliative care. The POS was originally developed to provide a multidimensional assessment of palliative care outcomes, including pain, emotional distress, family concerns, and existential well-being.

Among the individual domains, the highest scores were recorded for self-worth and the perception that life remained meaningful. These findings underscore the importance of existential and psychological dimensions in palliative care. Similar observations have been reported in studies using POS and IPOS, where emotional and existential concerns were strongly associated with overall quality of life and symptom burden [22]. Although many patients retained a sense of dignity and meaning, these domains were also the strongest contributors to the total POS score, suggesting that existential distress remains a central component of palliative care needs.

Pain and anxiety were also strongly correlated with the total POS score. This finding is consistent with previous studies showing that uncontrolled pain and emotional distress are among the most important determinants of poor palliative care outcomes and reduced quality of life [5,6,11]. Temel et al. [5] and Zimmermann et al. [6] demonstrated that early palliative care interventions targeting symptom control and psychological support significantly improved quality of life and mood in patients with advanced cancer. In addition, validation studies of the Integrated Palliative care Outcome Scale (IPOS) confirmed that physical and emotional symptoms were among the strongest contributors to overall symptom burden in patients with advanced illness [11,22,23]. These findings emphasize the importance of systematic pain assessment and the integration of psychological support into routine palliative care practice.

The POS questionnaire demonstrated moderate internal consistency, with a Cronbach’s alpha of 0.641. This value is comparable to findings from the Turkish validation study, in which the patient version of the POS showed a Cronbach’s alpha of 0.64 [13]. Recent psychometric studies of POS and IPOS have reported variable internal consistency across different cultural settings. For example, the Singaporean validation study by Long et al. demonstrated that language adaptation and cultural context may significantly influence internal consistency and construct validity, suggesting that psychometric performance may depend on language adaptation and population characteristics [24]. Although the observed reliability in this study is comparable to previous findings, it highlights the importance of further formal psychometric validation of the POS, including cultural adaptation and construct validation, before broader routine implementation in Kazakhstan [13].

No statistically significant differences in total POS scores were observed by gender or age group. Similar findings have been reported in several international studies, indicating that palliative care needs are driven more by disease severity and symptom burden than by basic demographic characteristics. This suggests that palliative care services should be organized according to patients’ clinical and psychosocial needs rather than demographic factors alone.

In contrast, significant differences were found across diagnostic groups. Patients with cancer reported the highest mean rank (89.91), whereas patients with dementia had the lowest mean rank (28.46). This may reflect differences in symptom burden, communication ability, and patterns of referral to palliative care. Differences across diagnostic groups may also reflect disease severity, functional status, cognitive impairment, and timing of palliative care referral, all of which may influence patient-reported outcomes. Previous studies have shown that patients with cancer often experience higher symptom burden, while non-cancer populations may follow different disease trajectories and have under-recognized palliative care needs [12,25]. Recent evidence also supports the applicability of integrated palliative outcome measures in non-cancer populations, confirming their relevance across diverse chronic and progressive conditions [26]. For example, Gao et al. [12] demonstrated substantial symptom burden among patients with progressive neurological conditions, while Grochowicka et al. [25] highlighted the broad applicability of the IPOS across diverse disease groups. Our regression analysis further suggested that disease profile may represent one of several factors associated with variation in total POS scores. However, the relatively low explained variance indicates that additional important determinants were not captured in the present model.

These findings have important implications for the development of palliative care in Kazakhstan. First, they support the potential integration of standardized patient-reported outcome measures such as the POS into palliative care assessment. Second, they highlight the importance of integrating psychological, existential, and symptom-focused interventions into clinical practice. Third, they indicate that diagnosis-specific approaches may be needed to better address the diverse needs of patients with cancer and non-cancer conditions.

Our findings are also consistent with recent evidence from Kazakhstan. Aimbetova et al. reported that both patients and nurses identified symptom management, psychosocial support, communication, and the care environment as key components of high-quality palliative care. The authors emphasized the importance of addressing not only physical symptoms but also emotional and supportive care needs to improve patient well-being. These observations align with the present study, in which anxiety, pain, self-worth, and meaning in life were among the most prominent concerns reported by patients receiving palliative care [27].

The study has several strengths. It is among the first quantitative investigations of patient-reported palliative care outcomes in Kazakhstan and applies a validated multidimensional instrument. The study also included patients with a wide range of diagnoses, providing a broader understanding of palliative care needs beyond oncology.

Several limitations should be acknowledged. First, the cross-sectional design precludes causal inference. Second, the study was conducted in one region of Kazakhstan and used convenience sampling, which may limit the generalizability of the findings. Third, some diagnostic subgroups included only a small number of participants, which may have affected the stability and interpretability of subgroup comparisons. Fourth, assisted questionnaire completion may have introduced response bias despite standardized administration procedures. Finally, the moderate internal consistency observed in this study, together with the absence of formal local validation, highlights the need for further psychometric evaluation of the POS in Kazakhstan.

Overall, this study demonstrates that patients receiving palliative care in Kazakhstan experience considerable physical, emotional, and existential concerns. Disease profile was one of the factors associated with variation in POS scores, while demographic characteristics showed limited influence. The findings suggest that the POS may be a useful tool for assessing multidimensional patient-reported outcomes in this setting; however, further formal psychometric validation is required before broader routine implementation in Kazakhstan.

## 5. Conclusions

This study demonstrated that patients receiving palliative care in the studied healthcare settings of the Abay region experience a moderate burden of physical, psychological, and existential concerns. Disease profile was significantly associated with patient-reported outcomes and may represent one of several factors influencing palliative care needs. The findings suggest that the Palliative Outcome Scale (POS) may be a useful tool for assessing multidimensional patient-reported outcomes in this setting. However, given the moderate internal consistency observed and the absence of formal psychometric validation in the Kazakhstani population, broader routine implementation should be approached cautiously. Future studies should focus on formal validation of the POS in Kazakhstan, longitudinal assessment of patient-reported outcomes, larger multicenter studies, comparisons between oncological and non-oncological populations, and evaluation of responsiveness to clinical change over time.

## Figures and Tables

**Table 1:** Sociodemographic and clinical characteristics of participants.

Variable	Category	n	%
Gender	Male	60	44.8
	Female	74	55.2
Age group	18–34 years	1	0.7
	35–49 years	14	10.4
	50–64 years	37	27.6
	65 years and older	82	61.2
Diagnosis	Dyscirculatory encephalopathy	34	25.4
	Cancer	27	20.1
	Stroke	25	18.7
	Parkinson’s disease	15	11.2
	Dementia	14	10.4
	Diabetes mellitus	13	9.7
	Bronchial asthma	4	3.0
	Syphilis	2	1.5
Questionnaire completion	Independently	37	27.6
	With the help of a friend or relative	36	26.9
	With the help of a staff member	61	45.5

**Table 2:** Descriptive statistics of POS items and total score.

POS Domain	Mean ± SD
Pain	1.21 ± 1.00
Nausea and constipation	0.62 ± 0.87
Anxiety and worry	1.43 ± 1.24
Family anxiety	0.96 ± 0.95
Information received	0.33 ± 0.79
Ability to share feelings with family	1.37 ± 1.42
Feeling that life is worthwhile	1.83 ± 1.22
Feeling good about oneself	1.89 ± 1.21
Time wasted due to healthcare visits	0.15 ± 0.53
Practical problems	0.34 ± 0.83
Total POS score	10.13 ± 5.05

Note: The total POS score ranges from 0 to 40. Higher scores indicate greater symptom burden, psychosocial distress, and unmet palliative care needs, whereas lower scores indicate fewer problems and better overall patient-reported outcomes.

**Table 3:** Reliability analysis of the POS questionnaire.

Indicator	Value
Cronbach’s alpha	0.641
Standardized Cronbach’s alpha	0.571
Number of items	10

**Table 4:** Spearman correlations between POS domains and total POS score.

Domain	Spearman’s r	*p*-Value
Feeling that life is worthwhile	0.775	<0.001
Feeling good about oneself	0.745	<0.001
Pain	0.618	<0.001
Anxiety and worry	0.598	<0.001
Ability to share feelings with family	0.530	<0.001
Family anxiety	0.355	<0.001
Nausea and constipation	0.313	<0.001
Practical problems	0.225	0.009
Time wasted due to healthcare visits	−0.185	0.032
Information received	0.159	0.066

**Table 5:** Mean ranks of total POS score by disease profile.

Disease Profile	n	Mean Rank
Dementia	14	28.46
Stroke	25	53.40
Dyscirculatory encephalopathy	34	67.51
Cancer	27	89.91
Bronchial asthma	4	74.50
Syphilis	2	84.00
Parkinson’s disease	15	74.77
Diabetes mellitus	13	77.00

**Table 6:** Comparison of Total POS scores by participant characteristics.

Variable	Statistical Test	Test Statistic	*p*-Value
Gender	Mann–Whitney U	U = 2018.5	0.366
Age group	Kruskal–Wallis	H = 1.321	0.724
Disease profile	Kruskal–Wallis	H = 28.356	<0.001

**Table 7:** Multiple linear regression analysis of factors associated with total POS score.

Predictor	B	β	95% CI	*p*-Value
Gender	1.073	0.106	−0.630 to 2.776	0.215
Age group	0.407	0.057	−0.775 to 1.589	0.497
Disease profile	0.688	0.292	0.278 to 1.097	0.001
Questionnaire completion method	0.650	0.108	−0.407 to 1.707	0.226

Model statistics: R^2^ = 0.092; Adjusted R^2^ = 0.064; F = 3.277; *p* = 0.014.

## Data Availability

The data presented in this study are available on request from the corresponding author due to privacy.

## Abbreviations

The following abbreviations are used in this manuscript:

IPOS	Integrated Palliative care Outcome Scale
POS	Palliative Outcome Scale
PROMs	Patient-Reported Outcome Measures
